# Molecular Surveillance of Coronaviruses in Riyadh (2025–2026): Persistent Genotype C and Conserved N-Glycosylation Motifs in Human Coronavirus OC43

**DOI:** 10.3390/ijms27083418

**Published:** 2026-04-10

**Authors:** Abdulrahman F. Alrezaihi, Ibrahim M. Aziz, Mohamed A. Farrag, Fahad M. Aldakheel, Abdulaziz M. Almuqrin, Lama Alzamil, Fuad Alanazi, Reem M. Aljowaie, Fahad N. Almajhdi

**Affiliations:** 1 Department of Clinical Laboratory Sciences, College of Applied Medical Sciences, King Saud University, Riyadh 12372, Saudi Arabiaaalmuqrin@ksu.edu.sa (A.M.A.); lalzamil@ksu.edu.sa (L.A.);; 2 Department of Botany and Microbiology, College of Science, King Saud University, Riyadh 11451, Saudi Arabia; iaziz@ksu.edu.sa (I.M.A.); mfarrag@ksu.edu.sa (M.A.F.); raljowaie@ksu.edu.sa (R.M.A.)

**Keywords:** molecular surveillance, HCoV-OC43, phylogenetic analysis, glycosylation sites

## Abstract

Seasonal human coronaviruses (HCoVs) continue to undergo adaptive evolution under structural and immune-mediated constraints. We investigated the molecular epidemiology and spike (S) protein structural variation of circulating coronaviruses in Riyadh, Saudi Arabia, during the 2025–2026 winter season, with particular emphasis on genotype persistence and glycosylation architecture in HCoV-OC43. Among 293 nasopharyngeal aspirates (NPAs) collected from hospitalized patients with acute respiratory illness, HCoV-OC43 was detected in 26 cases (8.87%), whereas other seasonal coronaviruses were not identified. Partial sequencing of the *S* gene revealed 97.84–98.23% nucleotide identity relative to the prototype strain VR-759, with amino acid substitutions distributed at discrete positions rather than within extended variable domains, indicating structural conservation. Phylogenetic reconstruction demonstrated that all Riyadh isolates clustered within genotype C, together with previously circulating local strains, supporting sustained endemic persistence and in situ evolution. In silico analysis of the S protein glycosylation landscape identified four invariant N-linked glycosylation motifs (N-X-S/T) at residues 46, 121, 134, and 190, reflecting strong structural constraints on glycan-dependent folding and antigenic configuration. A genotype-associated K68N substitution generated an additional N-glycosylation motif (68NGTD) in multiple Riyadh isolates, potentially modifying local glycan shielding without disrupting the overall glycosylation framework. The preservation of core glycosylation sites alongside selective motif acquisition suggests evolutionary fine-tuning of S surface topology rather than large-scale structural remodeling. Collectively, these findings indicate that genotype C persistence in Riyadh is accompanied by conserved S architecture and subtle glycosylation adjustments that may modulate immune recognition while maintaining structural integrity. Continued high-resolution molecular surveillance will be critical for defining the functional consequences of S microevolution in endemic HCoVs.

## 1. Introduction

Coronaviruses (CoVs) are a family of enveloped, positive-sense, single-stranded RNA viruses that are among the most common causes of respiratory infections in humans, ranging from the mild (common cold) to severe respiratory diseases such as pneumonia [1] CoVs possess the largest genomes among known RNA viruses, typically between 26–32 kilobases, and are characterized by distinctive spike (S) glycoproteins on their surface. These S projections, which give the virus its crown-like appearance (hence the name “corona”), mediate attachment to host cell receptors, enabling viral entry via direct fusion with the plasma membrane or endocytosis [1,2].

CoVs can infect a variety of animal species, which often act as reservoirs or intermediate hosts, facilitating zoonotic transmission to humans [2]. The genetic flexibility of CoVs allows them to continuously evolve, giving rise to novel strains capable of efficient human-to-human transmission [1,3].

Seven coronaviruses are known to infect humans: the seasonal human CoVs (HCoVs) 229E, NL63, OC43, and HKU1), as well as the highly pathogenic severe acute respiratory syndrome CoV (SARS-CoV), Middle East respiratory syndrome CoV (MERS-CoV), and SARS-CoV-2. Seasonal HCoVs are common causes of mild upper respiratory tract infections but can lead to more severe disease in vulnerable populations, such as young children, older adults, and immunocompromised individuals [4,5,6,7].

The repeated emergence of novel HCoVs highlights their considerable genetic plasticity and ongoing public health significance. Outbreaks of SARS, MERS, and coronavirus disease 2019 (COVID-19) have underscored the clinical and epidemiological importance of monitoring HCoVs, their evolution, and their impact on human health [8].

HCoV-OC43 is one of the most frequently detected seasonal HCoVs in clinical respiratory samples worldwide [9,10,11]. Its circulation is characterized by recurrent detection across consecutive epidemic seasons, with clear seasonal patterns observed over multiple years [12]. Molecular epidemiological studies demonstrate that HCoV-OC43 possesses significant genetic diversity, characterized by the simultaneous circulation of multiple genotypes and a clear pattern of lineage replacement over several decades [13,14]. This sustained circulation combined with ongoing genetic diversification makes HCoV-OC43 a suitable target for molecular epidemiological investigations aimed at understanding viral evolution and transmission dynamics.

Saudi Arabia experiences continuous population movement associated with international travel, workforce mobility, and large-scale religious activities, creating conditions that may facilitate the introduction and circulation of respiratory viruses [15]. These dynamics can accelerate viral importation, circulation, and mutation.

Although several local studies have described HCoV activity in previous years, comprehensive continuous molecular surveillance of HCoV-OC43 across multiple epidemic seasons in Saudi Arabia remains limited. Our earlier investigations provided snapshots of circulating strains during discrete periods (2016–2024) [16,17], but lacked longitudinal data on genotype persistence and structural evolution of the S protein. Given the continuous population movement associated with international travel, workforce mobility, and large-scale religious gatherings in Saudi Arabia, sustained molecular surveillance is essential to track importation, local evolution, and potential antigenic changes in endemic HCoVs. Therefore, the present study was conducted to (i) determine the prevalence and molecular epidemiology of seasonal coronaviruses in Riyadh during the 2025–2026 winter season, (ii) characterize genetic variation and genotype distribution of HCoV-OC43, and (iii) assess the conservation and microevolution of N-glycosylation motifs in the S protein, which are critical for folding, stability, and immune recognition. This longitudinal approach allows us to evaluate whether specific genotypes persist locally and whether subtle structural adaptations in the S glycoprotein are occurring under regional selective pressures.

## 2. Results

### 2.1. Prevalence of HCoV-OC43

HCoV-OC43 was found to be present in 26 cases out of 293 (8.87%) during the research period (winter 2025–2026). HCoV-OC43 infection was more prevalent in male patients (15 cases, 10.71%; *p* < 0.05) than in female patients (11 cases, 7.19%). Children between the ages of 5 and 14 had a significantly greater prevalence of HCoV-OC43 (10 cases, 12.82%; *p* < 0.05) than children in other age groups. Notably, HCoV-NL63, HCoV-HKU1, and HCoV-229E were not detected in any of the analyzed samples (Table 1).

### 2.2. Deduced Amino Acid Analysis of the S Protein

**Figure 1:**
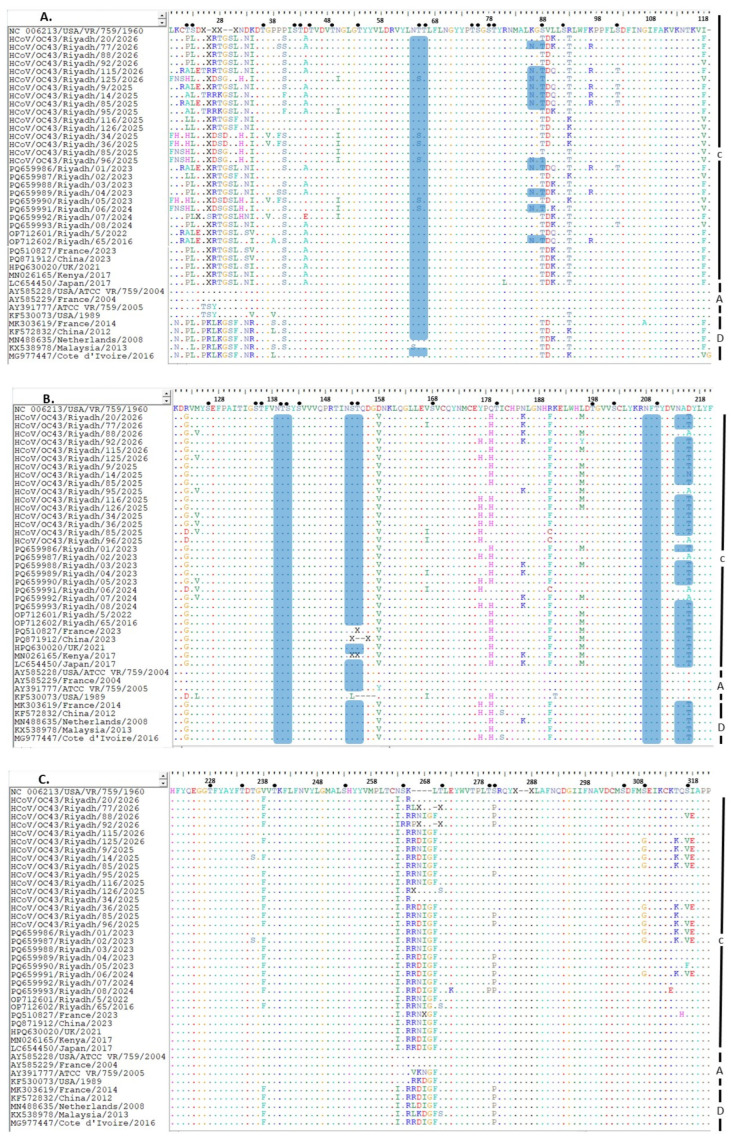
Deduced amino acid alignment of a 303-amino-acid fragment of the HCoV-OC43 S protein (corresponding to residues 19–321 relative to the prototype strain VR-759, NC_006213). Representative strains from each genotype, together with the Riyadh strains, were aligned using the ClustalW algorithm implemented in BioEdit software (version 7.0 The alignment is presented in three panels: (**A**) residues 19–119, (**B**) residues 120–220, and (**C**) residues 221–321. The prototype strain VR-759 (NC_006213), isolated in the USA in 1960, was used as the consensus reference sequence. Residues identical to the consensus are indicated by dots, whereas substitutions are shown as uppercase letters at their respective positions. Predicted O-glycosylation sites are marked with black dots, and potential N-glycosylation motifs (N-X-S/T) are highlighted in blue rectangles.

Partial sequencing of the *S* gene generated a 1086-nucleotide fragment that was translated into a 362-amino-acid polypeptide (residues 19–380 of the S protein). The Riyadh HCoV-OC43 strains showed 97.84–98.23% nucleotide identity in the *S* gene compared with the prototype strain VR-759 (USA, 1960). Alignment of the 362–amino acid S protein also demonstrated high overall conservation (Figure 1A–C). Most residues were identical to the consensus sequence, as demonstrated by continuous stretches of conserved positions across the 362 amino acid residues. Sequence variability was limited and occurred at discrete sites, with scattered amino acid substitutions rather than extensive regional divergence. These variations contributed to genotype differentiation, as certain substitutions were consistently shared within specific genotypes, indicating genotype-specific signature residues.

At the amino acid level, 50 of the 103-point mutations (48.54%) resulted in residue changes related to VR-759. Nineteen mutations (T4P/A or H, S5L, D15N, K16I, PT22S/F, S70T, V71D, R75T/K, I100F/V, R114G/D, D140V, Q161H, R172F/C, L178M, D198T/A, V220F, N245I, K247R, and L252F) were consistently detected in the majority of Riyadh and international strains. In addition, a sequence gap in the consensus strain at residues 8–12 was replaced by four amino acids (8R, 9T, 10G, and 12S), with a deletion at residue 13. Overall, the S protein exhibited limited but distinct variability sufficient for genotype discrimination while maintaining substantial structural conservation across strains.

### 2.3. Glycosylation Profile

The O- and N-glycosylation sites in the S protein of Riyadh and international strains were analyzed (Figure 1A–C). In silico prediction identified several conserved N-glycosylation motifs (N-X-S/T), highlighting their likely structural and functional relevance in maintaining proper protein folding, stability, and antigenic configuration. Four conserved N-glycosylation sites were consistently detected in both Riyadh and international strains at residues 46 (NTTL), 121 (NTSY), 134 (NSTQ), and 190 (NFTY), indicating strong evolutionary conservation of these glycan-associated regions.

Despite this overall conservation, minor genotype-specific differences were observed. Notably, a K68N substitution generated an additional potential N-glycosylation motif (68NGTD) in several Riyadh strains (77/2026, 115/2026, 125/2026, 9/2025, 14/2025, 85/2025, 96/2025) as well as in earlier strains (PQ659986/2023, PQ659989/2023, PQ659991/2024, and OP712602/2023). This site was absent in the analyzed international strains, suggesting a potentially region-specific evolutionary adaptation. The acquisition of this motif may influence local glycan shielding patterns and could contribute to immune evasion or altered receptor interactions.

Similarly, a D198T substitution resulted in the formation of an additional N-glycosylation motif (196NATY), which was observed in both Riyadh and international strains. The presence of this site across geographically distinct strains suggests that it may represent a broader evolutionary event rather than a localized adaptation.

In contrast, predicted O-linked glycosylation sites were more numerous, ranging from 38 to 40 sites among the analyzed strains. Although slight numerical variation was detected, the overall O-glycosylation pattern was highly comparable between Riyadh and international strains. Compatible O-glycosylation positions were consistently identified in all strains, indicating preservation of serine- and threonine-rich regions that may contribute to surface glycan density and antigenic masking.

Collectively, these findings demonstrate that while the S protein glycosylation landscape is largely conserved, the emergence of additional N-glycosylation motifs particularly the unique 68NGTD site in Riyadh strains may reflect ongoing microevolution and region-specific selective pressures.

### 2.4. Phylogenetic Analysis

**Figure 2:**
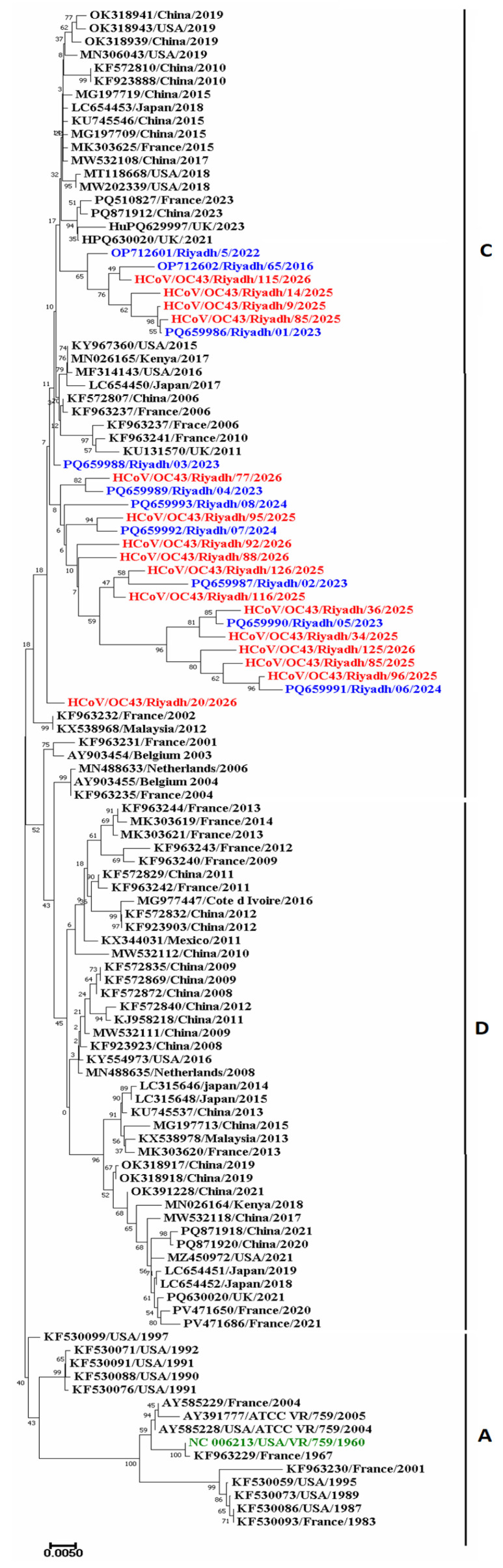
Phylogenetic analysis based on partial sequence of the *S* gene (1105nt). A total of 115 sequences were included in the phylogram for the *S* gene. A 1000 replicate bootstrapping value was used to assess the topology of the trees. Clustal W was used for multiple sequence alignment, and the evolutionary history was inferred by using the Neighbor-Joining method and Tamura-Nei model (MEGA X) to create the phylogram. Bootstrapping values below 50% were omitted from the phylogram. Riyadh strains of the winter season 2025/2026 are in red, local circulating strains in previous years are denoted in blue, and reference strains are shown in green.

The 26 Riyadh strains and 89 international strains were used to construct the phylogram (Figure 2). Based on the tree topology, the strains were initially clustered into three distinct genotypes (A, C, and D). The Riyadh strains from the winter season 2025/2026, together with earlier local Riyadh strains, consistently clustered within genotype C. This clustering pattern suggests sustained local evolution with limited divergence from previously circulating strains, while genotypes A and D comprised predominantly international reference strains.

## 3. Discussion

HCoVs are well established as leading causes of respiratory tract infections, producing a wide spectrum of clinical manifestations ranging from mild upper respiratory illness to more severe lower respiratory disease [18,19]. We previously investigated the molecular epidemiology and genetic evolution of circulating HCoVs subtypes in Riyadh, Saudi Arabia during the 2016–2024 seasons [16,17]. However, comprehensive data on the epidemiology and evolutionary dynamics of the four-common seasonal HCoVs (OC43-229E, NL63, and HKU1 remain limited in Saudi Arabia.

In the present study, we provide updated molecular insights into the circulation patterns, genetic variability, genotype distribution, and vaccine compatibility of HCoVs during the 2025/2026 winter season in Riyadh. Among 293 NPA samples collected from patients at KKUH, 26 cases (8.87%) tested positive for HCoV-OC43. All HCoV-OC43-positive cases were considered locally acquired infections within the Riyadh region; none of the patients had a recent history of international travel or known contact with travelers from other countries. Earlier surveillance (2016–2022) similarly identified HCoV-OC43 (4.15%) as the predominant strain, followed by HCoV-229E (1.1%) in Riyadh [19]. Furthermore, Hashem et al. (2019) detected HCoV-OC43 in respiratory samples from patients presenting with influenza-like illness, underscoring its contribution to seasonal respiratory infections in Saudi Arabia, particularly during the Hajj season [20]. Notably, HCoVs (NL63, HKU1, and 229E) were not detected in any of the samples analyzed. These findings are consistent with our previous report, which likewise documented no detection of these HCoVs types during the 2022/2023 and 2023/2024 winter seasons [17]. One possible explanation is viral interference, whereby infection with HCoV-OC43 may induce innate or cross-reactive immune responses that transiently suppress subsequent infection by other seasonal HCoVs [21,22]. Alternatively, the viral loads of HCoVs (NL63, HKU1, and 229E) in the study population may have been below the detection threshold of the diagnostic assays used, resulting in underestimation of their circulation. Differences in transmission dynamics, environmental stability, or temporal circulation patterns could also have contributed to their absence during the study period. Collectively, these findings emphasize the sustained predominance of HCoV-OC43 and its substantial contribution to the seasonal burden of viral respiratory illness in Saudi Arabia. Globally, HCoV-OC43 and HCoV-NL63 are generally the most prevalent seasonal HCoVs and are frequently encountered during early childhood infections [21]. The overall HCoV prevalence observed in our earlier study was 5.8%, with HCoV-OC43 (4.15%) being the most frequently detected virus, followed by HCoV-229E (1.1%). This distribution aligns with reports from China, the United Kingdom, and France [10,23,24,25]. For example, in China, analysis of 13,048 respiratory samples collected between 2010 and 2015 revealed an overall HCoVs prevalence of 2.25% (*n* = 294), with HCoV-OC43 accounting for 60.20% of cases, followed by HCoV-229E (16.67%), HCoV-NL63 (14.97%), and HCoV-HKU1 (7.82%) [19].

The HCoV-OC43 Riyadh strains showed 97.84–98.23% nucleotide identity in the *S* gene compared with the prototype HCoV-OC43 strain VR-759, with high amino acid conservation across the 362-aa *S* protein despite a 9.9% nucleotide mutation rate, consistent with our previous findings [17]. The relatively higher mutation rate in the *S* gene likely reflects immune-driven selective pressure, as the S protein mediates receptor binding and is a major target of neutralizing antibodies [26,27,28,29,30]. Such mutations may influence viral tropism, receptor affinity, antigenicity, and transmissibility. Although the full S protein comprises approximately 1353–1362 amino acids, the partial 362-aa S1 fragment analyzed here is sufficient for genotype assignment and evaluation of key glycosylation motifs while reflecting the region under strongest immune pressure.

Notably, deletions in the *S* gene were also reported in strains from France, the Netherlands, and China [31,32,33]. These findings highlight the evolutionary significance of *S* gene variation and underscore the importance of continued genomic surveillance to monitor emerging variants with potential public health implications [28,34,35].

Analysis of the S protein in Riyadh HCoV-OC43 strains identified nineteen amino acid substitutions, consistent with previously reported evolutionary patterns [16,17]. Additionally, a sequence gap in the consensus strain at residues 8–12 was replaced by four amino acids (8R, 9T, 10G, and 12S), accompanied by a deletion at residue 13. The clustering of mutations within specific regions (e.g., residues 19–25 and 310–317) may represent potential evolutionary hotspots [17]. Notably, several of these sites fall within domains implicated in receptor binding or immune evasion, highlighting their potential functional relevance. These observations align with findings from other regions of Saudi Arabia, where endemic strain evolution has been associated with seasonal dynamics and localized selective pressures. For instance, studies conducted in Jeddah reported amino acid identity differences of 26 ± 0.4% compared with strains from other geographic regions [36]. Overall, the S protein showed limited but distinct variability, sufficient for genotype differentiation while maintaining structural conservation. Mutation clustering in specific regions suggests possible evolutionary hotspots, particularly within domains related to receptor binding and immune evasion.

The HCoV-OC43 S protein uses 9-O-acetylated sialic acid (9-O-Ac-Sia) as its primary attachment receptor, with the binding site located in the N-terminal domain (NTD/S1A, approximately residues 15–300) [32,37,38]. The 362-amino-acid fragment sequenced in this study fully covers this domain. None of the 19 conserved amino acid substitutions identified in the Riyadh genotype C isolates (T4P/A or H, S5L, D15N, K16I, PT22S/F, S70T, V71D, R75T/K, I100F/V, R114G/D, D140V, Q161H, R172F/C, L178M, D198T/A, V220F, N245I, K247R, and L252F) map directly into the core 9-O-Ac-Sia binding pocket. However, several substitutions (particularly around residues 70–75 and 100–114) lie in surface loops flanking the receptor-binding site and could subtly modulate binding affinity, glycan accessibility, or antigenic presentation.

Notably, the genotype-associated K68N substitution, which creates an additional N-glycosylation motif (68NGTD) in multiple Riyadh isolates, is located in close proximity to the receptor-binding groove. Acquisition of this N-linked glycan may provide additional shielding of the NTD surface, potentially contributing to immune evasion or fine-tuning sialic acid interactions without disrupting the core receptor-binding architecture [38]. This localized microevolution is consistent with the overall conservation of the four invariant N-glycosylation sites and supports gradual adaptation under immune pressure while preserving essential receptor engagement.

The S protein of HCoV-OC43 is a heavily glycosylated class I fusion protein, harboring approximately 22 potential N-linked glycosylation sites per protomer that are essential for proper folding, structural stability, and immune evasion [39]. Our analysis demonstrates that, despite ongoing viral evolution, glycosylation patterns remain largely conserved between Riyadh and international strains. The S protein exhibited comparable O-glycosylation profiles (38–40 sites) and four invariant N-glycosylation motifs (residues 46, 121, 134, and 190), underscoring strong structural constraints on key functional domains involved in receptor binding and membrane fusion. This conservation suggests that while sequence-level mutations accumulate, glycan-associated regions remain selectively preserved to maintain viral fitness and host adaptability. However, minor genotype-specific variations were detected. The K68N substitution, which introduced an additional N-glycosylation motif (68NGTD) in multiple Riyadh strains, may represent a region-specific adaptation potentially affecting glycan shielding, immune evasion, or receptor affinity. Conversely, the D198T substitution (196NATY) was present in both Riyadh and international strains, suggesting a broader evolutionary advantage rather than a localized adaptation. These observations underscore the role of subtle glycosylation changes in modulating viral antigenicity and host interactions [40].

The genetic diversity of HCoV-OC43 has been well documented, with initial studies identifying four genotypes (A–D), of which genotype D arose through recombination [14,41]. More recent whole-genome analyses have proposed up to 11 genotypes (A–K), with genotypes G, J, and K becoming increasingly dominant globally in recent years [references if available, e.g., recent papers from 2022–2025]. In the present study, all Riyadh isolates clustered within genotype C together with previously circulating local strains from 2016–2024, indicating sustained endemic persistence of this lineage in Saudi Arabia rather than replacement by newer genotypes (G, J, or K). This regional pattern may reflect local selective pressures or limited importation of emerging lineages during the 2025–2026 winter season.

All Riyadh isolates from the 2025/2026 winter season were grouped within genotype C, together with previously circulating local strains from the 2016–2024 seasons [16,17]. This pattern suggests sustained local evolution with limited divergence, rather than repeated introduction of novel external lineages. The genetic variability observed within genotype C likely reflects localized selective pressures and potential recombination events—well-recognized mechanisms contributing to HCoV-OC43 diversity. In contrast, genotypes A and D consisted predominantly of international reference strains, indicating geographically structured lineage distribution.

Notably, the evolutionary trees also revealed close relationships between Riyadh and international strains, particularly within genotype C, suggesting interregional transmission pathways likely facilitated by human mobility. The phylogenetic proximity of Riyadh strains to European and Asian isolates supports a cosmopolitan dispersal pattern. Similar transmission dynamics have been reported in studies tracking global circulation and zoonotic reservoirs of HCoV-OC43 [14,42], further underscoring the virus’s genetic plasticity and capacity for widespread geographic dissemination [14,25,29].

This study has several limitations. First, the samples were collected from a single hospital (KKUH) in Riyadh, which may not fully represent the broader population, including potential geographic and demographic variability. Second, only partial sequencing of the *S* gene was performed, limiting the ability to detect recombination events and fully characterize viral genomic diversity. Whole-genome sequencing would provide a more comprehensive understanding of viral evolution. Third, comparative structural analysis with SARS-CoV-2 S protein may provide additional evolutionary insights and will be considered in future studies. Finally, coinfections with other respiratory viruses, including SARS-CoV-2, were not evaluated and warrant further investigation.

## 4. Materials and Methods

### 4.1. Clinical Samples and Ethics Statement

A total of 293 NPA samples were collected from hospitalized patients at King Khalid University Hospital (KKUH) in Riyadh between November 2025 and February 2026, corresponding to the winter respiratory virus season. Patients were included based on acute respiratory symptoms, such as fever, cough, dyspnoea, and rhinorrhea. The study was conducted following the Declaration of Helsinki and was approved by the King Saud University Institutional Review Board Ethics Committee in Riyadh, Saudi Arabia (Ethics Reference No. E-259942/IRB), approved in July 2025. Written informed consent was obtained from the parents or guardians of all participants prior to sample collection. Samples were transported in a viral transport medium (MEM) and stored at −80 °C until processing.

### 4.2. Viral RNA Extraction and Screening

Viral RNA was extracted from the NPA samples using the QIAamp viral RNA extraction kit (Qiagen, Hilden, Germany) following the manufacturer’s instructions. Initial screening for the four common HCoVs (HCoV-OC43, -NL63, -HKU1, and -229E) was performed using the OneStep RT-PCR kit (Qiagen). Screening was performed using previously described pan-coronavirus primers, as reported in our earlier studies [17]. Reverse transcription and PCR amplification were carried out under standard conditions, including an initial reverse transcription step, enzyme activation, and repeated cycles of denaturation, annealing, and extension, as previously described.

### 4.3. Detection, Typing, and Amplification of the S Gene

Samples that tested positive during screening were subjected to typing reactions using specific primer sets for HCoV-OC43, -NL63, -HKU1, and -229E. Samples were not tested for SARS-CoV-2 or other non-coronavirus respiratory viruses using multiplex panels. For positive HCoV-OC43 samples, partial sequencing of the *S* gene generated a 1086-nucleotide fragment and was amplified using the SuperScript III or One-Step Ahead RT-PCR System with high-fidelity DNA polymerase. PCR products were visualized using 1% ethidium-bromide-stained agarose gel electrophoresis.

### 4.4. Nucleotide and Amino Acid Sequencing Analysis

Purified *S* gene fragments were sequenced on both strands. Raw sequence data were edited and assembled using Bioedit software (version 7.0). Multiple sequence alignments were performed using the Clustal W algorithm within the MegAlign program. To identify mutations and determine genotypes, sequences were compared against international reference strains retrieved from GenBank, with the prototype strain VR-759 (NC_006213) serving as a consensus sequence. The genes’ final sequences were deposited in the gene bank database under the accession number PZ024089-PZ024103.

### 4.5. Phylogenetic Analysis and Genotype Assignment

Phylogenetic trees were constructed using the Maximum Likelihood (ML) method in MEGA (version X), with branch support assessed by 1000 bootstrap resampling iterations. To ensure rigid genotype assignment, p-distance values were calculated to evaluate intra-genotype and inter-genotype distances, using established thresholds to categorize circulating Riyadh strains. Genotype assignment was based on phylogenetic clustering and p-distance analysis using partial *S* gene sequences. Although up to 11 genotypes (A–K) have been proposed in recent whole-genome analyses, the partial S fragment used here reliably resolves genotypes A, C, and D.

## 5. Conclusions

This study provides updated molecular epidemiological data on HCoV-OC43 circulating in Riyadh during the 2025–2026 winter season. HCoV-OC43 was the only seasonal coronavirus detected and accounted for 8.87% of respiratory cases, confirming its continued predominance in the region.

Genetic analysis of the *S* gene demonstrated high nucleotide and amino acid conservation relative to the prototype strain Human coronavirus OC43, with limited but distinct genotype-specific substitutions. The conservation of key N-glycosylation motifs underscores structural constraints on S protein function, whereas the emergence of an additional glycosylation motif (68NGTD) in several Riyadh strains may reflect localized adaptive evolution.

Phylogenetic findings showed that all contemporary Riyadh strains cluster within genotype C, together with strains from previous local seasons, suggesting sustained endemic circulation and in situ evolution rather than frequent lineage replacement. These findings highlight the dynamic yet structured evolutionary behavior of HCoV-OC43 in Saudi Arabia.

Continued long-term molecular surveillance, ideally incorporating whole-genome sequencing and functional analyses, is essential to better understand evolutionary trends, recombination events, and potential antigenic changes that may influence viral transmission, immune escape, and public health impact.

## Figures and Tables

**Table 1:** Sample distribution across epidemic seasons, gender, and age groups.

		No. of Samples	Positive for HCoV-			
			OC43	229E	NL63	HKU1
		*n*	*n* (%)	*n* (%)	*n* (%)	*n* (%)
Season	2025	196 (66.89)	20 (10.20)	0	0	0
	2026	97 (33.11)	6 (6.19)	0	0	0
	Total	293	26 (8.87)	0	0	0
Gender	Male	140 (47.78)	15 (10.71) ^a^	0	0	0
	Female	153 (52.22)	11 (7.19)	0	0	0
Age in years	0–4	88 (30.03)	5 (5.68)	0	0	0
	5–14	78 (26.62)	10 (12.82) ^b^	0	0	0
	15–64	71 (24.23)	7 (9.86)	0	0	0
	≥65	56 (19.1)	4 (7.14)	0	0	0

Data are displayed as percentages (%). Comparisons between groups were performed using Chi-square test (SPP). ^a^ Significant and indicates a significantly greater value (*p* < 0.05) compared to female. ^b^ Significantly different (*p* < 0.05) from age groups 0–4, 15–64, and ≥65 years.

## Data Availability

The datasets generated and analyzed during the current study are available from the corresponding author upon reasonable request. The sequence data reported in this study have been deposited in GenBank under accession numbers PZ024089-PZ024103.

## References

[1] Coerdt K.M., Khachemoune A. (2021). Corona viruses: Reaching far beyond the common cold. Afr. Health Sci..

[2] Greenberg S.B. (2016). Update on Human Rhinovirus and Coronavirus Infections. Semin. Respir. Crit. Care Med..

[3] Su S., Wong G., Shi W., Liu J., Lai A.C.K., Zhou J., Liu W., Bi Y., Gao G.F. (2016). Epidemiology, Genetic Recombination, and Pathogenesis of Coronaviruses. Trends Microbiol..

[4] Wevers B.A., van der Hoek L. (2009). Recently discovered human coronaviruses. Clin. Lab. Med..

[5] Farrag M.A., Amer H.M., Bhat R., Hamed M.E., Aziz I.M., Mubarak A., Dawoud T.M., Almalki S.G., Alghofaili F., Alnemare A.K. (2021). SARS-CoV-2: An overview of virus genetics, transmission, and immunopathogenesis. Int. J. Environ. Res. Public Health.

[6] V’kovski P., Kratzel A., Steiner S., Stalder H., Thiel V. (2021). Coronavirus biology and replication: Implications for SARS-CoV-2. Nat. Rev. Microbiol..

[7] Bashatwah R.M., Aljabali A.A., Tambuwala M.M. (2024). SARS-CoV-2 Variants and Global Vulnerability: Diagnostic, Vaccines, and Therapeutic Management. SARS-CoV-2 Variants and Global Population Vulnerability.

[8] Izhari M.A., Alghamdi F., Alodeani E.A., Salem A.A., Almontasheri A.H.A., Dardari D.M.M., Hadadi M.A.A., Gosady A.R.A., Alghamdi W.A., Alzahrani B.A. (2025). Evolutionary Insight into Fatal Human Coronaviruses (hCoVs) with a Focus on Circulating SARS-CoV-2 Variants Under Monitoring (VUMs). Biomedicines.

[9] Liu D.X., Liang J.Q., Fung T.S. (2021). Human coronavirus-229E,-OC43,-NL63, and-HKU1 (Coronaviridae). Encycl. Virol..

[10] Gaunt E.R., Hardie A., Claas E.C., Simmonds P., Templeton K.E. (2010). Epidemiology and clinical presentations of the four human coronaviruses 229E, HKU1, NL63, and OC43 detected over 3 years using a novel multiplex real-time PCR method. J. Clin. Microbiol..

[11] Kovacs D., Mambule I., Read J.M., Kiran A., Chilombe M., Bvumbwe T., Aston S., Menyere M., Masina M., Kamzati M. (2024). Epidemiology of Human Seasonal Coronaviruses Among People with Mild and Severe Acute Respiratory Illness in Blantyre, Malawi, 2011–2017. J. Infect. Dis..

[12] Wilson R., Kovacs D., Crosby M., Ho A. (2024). Global Epidemiology and Seasonality of Human Seasonal Coronaviruses: A Systematic Review. Open Forum Infect. Dis..

[13] Vijgen L., Keyaerts E., Moës E., Thoelen I., Wollants E., Lemey P., Vandamme A.M., Van Ranst M. (2005). Complete genomic sequence of human coronavirus OC43: Molecular clock analysis suggests a relatively recent zoonotic coronavirus transmission event. J. Virol..

[14] Lau S.K., Lee P., Tsang A.K., Yip C.C., Tse H., Lee R.A., So L.Y., Lau Y.L., Chan K.H., Woo P.C. (2011). Molecular epidemiology of human coronavirus OC43 reveals evolution of different genotypes over time and recent emergence of a novel genotype due to natural recombination. J. Virol..

[15] Farrag M.A., Hamed M.E., Amer H.M., Almajhdi F.N. (2019). Epidemiology of respiratory viruses in Saudi Arabia: Toward a complete picture. Arch. Virol..

[16] Alamri K.A., Farrag M.A., Aziz I.M., Dudin G.A., Mohammed A.A., Almajhdi F.N. (2022). Prevalence of human coronaviruses in children and phylogenetic analysis of HCoV-OC43 during 2016–2022 in Riyadh, Saudi Arabia. Viruses.

[17] Aljowaie R.M., Farrag M.A., Aziz I.M., Alkubaisi N.A., Alzayed R.M., Alhajouj S.A., Aldosari N.S., Alhetheel A.F., Almajhdi F.N. (2025). Human coronavirus OC43 in Saudi Arabia: Molecular epidemiology and prevalence in hospitalized children during 2022–24. Future Virol..

[18] Fischer N., Dauby N., Bossuyt N., Reynders M., Gerard M., Lacor P., Daelemans S., Lissoir B., Holemans X., Magerman K. (2021). Monitoring of human coronaviruses in Belgian primary care and hospitals, 2015–2020: A surveillance study. Lancet Microb..

[19] Zhang S.F., Tuo J.L., Huang X.B., Zhu X., Zhang D.M., Zhou K., Yuan L., Luo H.J., Zheng B.J., Yuen K.Y. (2018). Epidemiology characteristics of human coronaviruses in patients with respiratory infection symptoms and phylogenetic analysis of HCoV-OC43 during 2010–2015 in Guangzhou. PLoS ONE.

[20] Hashem A.M., Al-Subhi T.L., Badroon N.A., Hassan A.M., Bajrai L.H.M., Banassir T.M., Alquthami K.M., Azhar E.I. (2019). MERS-CoV, influenza and other respiratory viruses among symptomatic pilgrims during 2014 hajj season. J. Med. Virol..

[21] Dijkman R., Jebbink M.F., Gaunt E., Rossen J.W., Templeton K.E., Kuijpers T.W., Van der Hoek L. (2012). The dominance of human coronavirus OC43 and NL63 infections in infants. J. Clin. Virol..

[22] Kong D., Zheng Y., Hu L., Chen J., Wu H., Teng Z., Zhou Y., Qiu Q., Lu Y., Pan H. (2021). Epidemiological and co-infection characteristics of common human coronaviruses in Shanghai, 2015–2020: A retrospective observational study. Emerg. Microbes Infect..

[23] Lau S.K., Woo P.C., Yip C.C., Tse H., Tsoi H.W., Cheng V.C., Lee P., Tang B.S., Cheung C.H., Lee R.A. (2006). Coronavirus HKU1 and other coronavirus infections in Hong Kong. J. Clin. Microbiol..

[24] Masse S., Capai L., Villechenaud N., Blanchon T., Charrel R., Falchi A. (2020). Epidemiology and Clinical Symptoms Related to Seasonal Coronavirus Identified in Patients with Acute Respiratory Infections Consulting in Primary Care over Six Influenza Seasons (2014–2020) in France. Viruses.

[25] Zhang Y., Li J., Xiao Y., Zhang J., Wang Y., Chen L., Paranhos-Baccala G., Ren L., Wang J. (2015). Genotype shift in human coronavirus OC43 and emergence of a novel genotype by natural recombination. J. Infect..

[26] Du L., He Y., Zhou Y., Liu S., Zheng B.-J., Jiang S. (2009). The spike protein of SARS-CoV—A target for vaccine and therapeutic development. Nat. Rev. Microbiol..

[27] Premkumar L., Segovia-Chumbez B., Jadi R., Martinez D.R., Raut R., Markmann A.J., Cornaby C., Bartelt L., Weiss S., Park Y. (2020). The receptor-binding domain of the viral spike protein is an immunodominant and highly specific target of antibodies in SARS-CoV-2 patients. Sci. Immunol..

[28] Ren L., Zhang Y., Li J., Xiao Y., Zhang J., Wang Y., Chen L., Paranhos-Baccalà G., Wang J. (2015). Genetic drift of human coronavirus OC43 spike gene during adaptive evolution. Sci. Rep..

[29] Forni D., Cagliani R., Arrigoni F., Benvenuti M., Mozzi A., Pozzoli U., Clerici M., De Gioia L., Sironi M. (2021). Adaptation of the endemic coronaviruses HCoV-OC43 and HCoV-229E to the human host. Virus Evol..

[30] Jo W.K., Drosten C., Drexler J.F. (2021). The evolutionary dynamics of endemic human coronaviruses. Virus Evol..

[31] Kin N., Miszczak F., Lin W., Ar Gouilh M., Vabret A., Consortium E. (2015). Genomic analysis of 15 human coronaviruses OC43 (HCoV-OC43s) circulating in France from 2001 to 2013 reveals a high intra-specific diversity with new recombinant genotypes. Viruses.

[32] Wang C., Hesketh E.L., Shamorkina T.M., Li W., Franken P.J., Drabek D., van Haperen R., Townend S., van Kuppeveld F.J., Grosveld F. (2022). Antigenic structure of the human coronavirus OC43 spike reveals exposed and occluded neutralizing epitopes. Nat. Commun..

[33] Shao N., Zhang C., Dong J., Sun L., Chen X., Xie Z., Xu B., An S., Zhang T., Yang F. (2022). Molecular evolution of human coronavirus-NL63,-229E,-HKU1 and-OC43 in hospitalized children in China. Front. Microbiol..

[34] Bermudez Y., Miles J., Muller M. (2023). Nonstructural protein 1 widespread RNA decay phenotype varies among coronaviruses. Iscience.

[35] Song W., Fang Z., Ma F., Li J., Huang Z., Zhang Y., Li J., Chen K. (2023). The role of SARS-CoV-2 N protein in diagnosis and vaccination in the context of emerging variants: Present status and prospects. Front. Microbiol..

[36] Sohrab S.S., Alsaqaf F., Hassan A.M., Tolah A.M., Bajrai L.H., Azhar E.I. (2024). Genomic Diversity and Recombination Analysis of the Spike Protein Gene from Selected Human Coronaviruses. Biology.

[37] Hulswit R.J.G., Lang Y., Bakkers M.J.G., Li W., Li Z., Schouten A., Ophorst B., van Kuppeveld F.J.M., Boons G.J., Bosch B.J. (2019). Human coronaviruses OC43 and HKU1 bind to 9-O-acetylated sialic acids via a conserved receptor-binding site in spike protein domain A. Proc. Natl. Acad. Sci. USA.

[38] Tortorici M.A., Walls A.C., Lang Y., Wang C., Li Z., Koerhuis D., Boons G.J., Bosch B.J., Rey F.A., de Groot R.J. (2019). Structural basis for human coronavirus attachment to sialic acid receptors. Nat. Struct. Mol. Biol..

[39] Mounir S., Talbot P.J. (1993). Molecular characterization of the S protein gene of human coronavirus OC43. J. Gen. Virol..

[40] Ming A., Zhao J., Liu Y., Wang Y., Wang X., Li J., Zhang L. (2024). O-glycosylation in viruses: A sweet tango. Mlife.

[41] Vijgen L., Keyaerts E., Lemey P., Moës E., Li S., Vandamme A.M., Van Ranst M. (2005). Circulation of genetically distinct contemporary human coronavirus OC43 strains. Virology.

[42] Kayode A.J., Banji-Onisile F.O., Olaniran A.O., Okoh A.I. (2021). An overview of the pathogenesis, transmission, diagnosis, and management of endemic human coronaviruses: A reflection on the past and present episodes and possible future outbreaks. Pathogens.

